# Feasibility of a nurse-led, mHealth-assisted, and team-based collaborative care model for heart failure care in India: Findings from a multi-stakeholder qualitative study

**DOI:** 10.12688/wellcomeopenres.21175.2

**Published:** 2024-10-15

**Authors:** Sunu C. Thomas, Kandagathuparambil Neenumol, Susanna Chacko, Jose Prinu, Meera R. Pillai, Sunil Pisharody, Somanathan Chozhakkat, MS Jyothi Vijay, A. Mohamed Iliyas, Sivadasanpillai Harikrishnan, Sanjay Ganapathi, Panniyammakal Jeemon

**Affiliations:** 1 Sree Chitra Tirunal Institute for Medical Sciences and Technology, Trivandrum, Kerala, 695011, India; 2 KIMS Hospital, Thiruvananthapuram, Kerala, India; 3 EMS Memorial Cooperative Hospital and Research Centre Ltd, Malappuram, Kerala, India

**Keywords:** heart failure, collaborative care model, team-based, nurse-led, India, TIME-HF

## Abstract

**Background:**

Heart failure (HF) management is often challenging due to poor adherence to GDMT and self-care. Continuous monitoring of patients by a dedicated care manager may enhance adherence to self-care and treatment and prevent hospitalisations. For the adoption and acceptance of a collaborative care model (CCM) for HF management in Indian settings, understanding the perspectives of all stakeholders regarding its various components and feasibility is needed. Therefore, we aimed to obtain perceptions of potential challenges to care and suggestions on multiple components of the proposed CCM in managing HF and its feasibility.

**Methods:**

In-depth interviews were done among HF patients, caregivers, nurses, and cardiologists from private, co-operative, and public sector tertiary care hospital settings that cater to HF patients in Kerala, India. An in-depth interview guide was used to elicit the data. Data were analysed using Python QualCoder version 2.2. We used a framework method for the analysis of data.

**Results:**

A total of 22 in-depth interviews were conducted. We found that the existing care for HF in many settings was inadequate for continuous engagement with the patients. Non-adherence to treatment and other self-care measures, was noted as a major challenge to HF care. Healthcare providers and patients felt nurses were better at leading collaborative care. However, various barriers, including technical and technological, and the apprehensions of nurses in leading the CCM were identified. The stakeholders also identified the mHealth-assisted CCM as a potential tool to save money. The stakeholders also appreciated the role of nurses in creating confidence in patients.

**Conclusions:**

A nurse-led, mHealth-assisted, and team-based collaborative care was recognised as an excellent step to improve patient adherence. Effective implementation of it could reduce hospitalisations and improve patients' ability to manage their HF symptoms.

## Plain language summary

Heart failure (HF) management requires continuous monitoring of patients by a dedicated care manager to improve adherence to self-care and treatment and prevent hospitalisations. In this study, we aimed to obtain perceptions of patients, carers, cardiologist and nurses on the current challenges to HF care. We also elicited the feasibility of a proposed nurse-led team based collaborative care model in managing HF with mHealth assistance and their suggestions on various components in the model. By interviewing 22 heart failure stakeholders we found the various individual, system level challenges in HF care. Non-adherence to treatment and other self-care measures, was mentioned as a major challenge to HF care. To address the challenges, healthcare providers and patients felt nurses can lead the collaborative care. The stakeholders also identified the mHealth-assisted collaborative care model (CCM) can save money by avoiding unnecessary travel for the patients. While the stakeholders appreciated the role of nurses in creating confidence in patients, they also highlighted the challenges in implementing the intervention. In order to overcome these challenges, training nurses on the basics of HF medications, their side effects, and contraindications was suggested. The stakeholders also mentioned various technical and technological barriers in the use of mHealth application.

## Introduction

Heart failure (HF) is becoming an increasingly concerning global public health issue ^1^. Although the prevailing guideline-directed medical therapies (GDMT) along with behavioural modification strategies significantly improve mortality outcomes in HF patients, adherence to these therapies remains a significant public health concern ^2– 4^. Despite the available evidence on survival benefits, only one in four eligible patients receive GDMT in India ^5– 8^. Heart failure management that focuses on optimising therapy, improving self-care through patient education, frequent follow-up, and continuous monitoring for early identification of signs and symptoms of worsening HF are important in limiting the number of re-hospitalizations ^9^.

In India, managing HF is often challenging due to poor adherence to GDMT and self-care, which is caused by a lack of motivation and the belief that treatment is burdensome. Continuous and positive reinforcement, aided by real-time monitoring from a dedicated care manager, can enhance adherence ^10^. A physician-driven disease management program may be challenging due to the high patient load in low- and middle-income countries (LMIC) ^11^. However, task-sharing strategies involving nurses and other cadres of healthcare providers are acceptable and effective in managing hypertension and diabetes in LMICs ^12, 13^. In addition, collaborative care models (CCM) involving nurses acting as care coordinators effectively manage HF in high-income countries ^9^. Studies have shown that nurse-led management in HF with regular follow-up and patient education have positive impact on patients with improved compliance, quality of life as well as increased survival and decreased number of hospitalisations ^14– 17^. The goal of CCM is to merge service delivery and involve various healthcare providers, such as cardiologists, nurses, and other specialists, to provide the best possible and coordinated care for patients with HF. In CCM, nurses frequently serve as care coordinators and ensure that patient-centred care is provided when managing HF. However, these models have not been tested in Indian settings to determine their effectiveness in improving survival outcomes and quality of life for patients with HF. We are developing a team-based collaborative care model (CCM), facilitated by trained nurses, for management of HF in India. We conceptualised team-based collaborative care model as team-based approach to HF care with nurses as a key team player ^18^.

To improve the adoption and acceptance of the CCM for HF management in Indian settings, it is essential to gather and consider the perspectives and opinions of all stakeholders regarding its various components, such as lifestyle education, disease management programs, pharmacologic treatment, self-care, and care coordination. This will help tailor the model to meet their needs and preferences better. Gaining a thorough understanding of the viewpoints of those affected by heart failure - patients, caregivers, and healthcare providers - can aid in recognising obstacles and implementing CCM for managing the condition. Therefore, we aimed to involve patients, carers, nurses, and cardiologists in a qualitative research study to obtain critical insights into their experiences, perceptions of potential challenges to care, and suggestions on various components of the proposed CCM in managing HF. The use of digital technologies makes it easier to put CCM into practice. These technologies can support patient screening, referral, monitoring, and treatment ^19^. Mobile applications have reportedly increased the efficiency of monitoring patient reported outcomes especially in patients with chronic conditions including cardiovascular diseases, who require continuous monitoring by healthcare providers. It has also been reported that these apps can help patients with chronic conditions to have greater improvements in their satisfaction levels to decreased complications and fewer hospital admissions ^19– 21^. In the CCM, we have an mHealth component with two linked application one for patients and another for nurses, which is developed exclusively for the intervention. The details of the mHealth intervention are explained in the protocol paper ^18^. The mHealth patient application is available in Google Play.

Therefore, the aim of this study was to obtain perceptions of potential challenges to heart failure care and suggestions on multiple components of the proposed CCM in managing HF and its feasibility. Since this model envisaged a nurse-facilitated CCM, we also were looking for the acceptability of nurses in leading this collaborative care. Additionally, we sought to obtain feedback from stakeholders regarding various aspects of the mHealth model. The findings from the study can help tailor the team-based CCM model (TIME-HF) to adapt it to the cultural context.

## Methods

### Ethical consideration

The research study is approved by institutional ethics committee of Sree Chitra Tirunal Institute for Medical Sciences and Technology (SCTIMST) and of the participating centres (SCT/IEC/1691/AUGUST-2021). Written informed consent was obtained from all the participants before starting the data collection. All the identifying information of the participants are anonymised.

### Study setting

The study was conducted in private, co-operative, and public sector tertiary care hospital settings that cater to HF patients in Kerala, India. Kerala’s healthcare system encompasses both public and private sectors, with most hospitals and beds in the private sector ^22^. The average total medical expenditure per hospitalisation for cardio-vascular diseases is eight times higher in private-sector hospitals compared to public-sector hospitals ^23^. Both public and private tertiary care hospitals manage HF, where cardiologists are primary providers, with referrals to other departments depending on the patient’s underlying chronic conditions.

We identified stakeholders from three private, one co-operative, and one public sector tertiary care hospital, based on their experience and expertise in heart failure management. These hospitals are multi-specialty centres with facilities for managing HF, with one of them exclusively focusing on cardiovascular and neurological conditions. The total bed capacity of the chosen hospitals ranged from 250 to 1100.

### Study participants, recruitment, and sampling

Study participants were various HF care stakeholders, such as patients, caregivers, and healthcare providers (HCPs) like nurses and cardiologists. We conducted in-depth interviews with all of them. We purposively selected cardiologists and nurses with experience in HF care from participating centres of a large cluster randomized trial ^18^. These centres were chosen based on whether they had dedicated HF clinics or not. In centres with HF clinics, patients received regular follow-ups from dedicated nurses, while in other centres, cardiologists managed patients in outpatient settings. To recruit patients and their caregivers, we used purposive sampling. We approached nurses working in HF clinics or cardiology departments for patient and caregiver recruitment. Eligible participants were adults aged 18 years or older with an HF diagnosis. We excluded patients with acute symptoms and those awaiting outpatient department consultations. Caregivers accompanying patients to the chosen hospitals' outpatient settings were also included.

### Data collection, quality control and management

Data were collected from May to June 2022 by a post-doctoral level researcher (SCT) with assistance from two research fellows with post-graduate qualifications (NKR & SC), all of whom were females. Data were collected using semi-structured, in-depth interview guides. All the interviews were audio recorded with permission from the participants. The interview guides for patients and caregivers were translated into the regional language, Malayalam. All data collection instruments were semi-structured guides tailored to the nature of the selected stakeholder and designed to elicit information on the perceived quality of barriers, facilitators to HF management, and specific feedback on the proposed CCM ^18^. Patients' and caregivers' interest in a mHealth-assisted program and acceptability of intervention strategies were also explored. The interview questions and prompts were developed based on the proposed team-based CCM ^18^ as well as relevant literature on collaborative care models and its perceived barriers and facilitators ^24– 27^. Initial interviews explored essential questions from the guide to the chosen participants. Afterward, we slightly modified the interview guide to thoroughly investigate specific domains or areas brought up during the initial interviews. The interview guides are given as *Extended data* ^28^.

We interviewed patients, caregivers, and nurses in Malayalam and opted for English when interviewing cardiologists to facilitate a more comprehensive discussion. On an average the interviews ranged from 30–45 minute, with majority lasted for 30 minutes, while a few interviews with cardiologists and nurses, lasted between 30–45 minutes. Considering the convenience of some patients and caregivers, we conducted in-depth interviews with selected participants in the comfort of their own homes. Written informed consent from all the participants was obtained before the interview, and de-identified all information to ensure confidentiality. The Consolidated Criteria for Reporting Qualitative Research Standards (COREQ)- a 32 item checklist was used to guide the reporting of this study ^29^.

### Data analysis

A post-doctoral fellow carefully reviewed the full transcripts while listening to the audio recordings to ensure the accuracy and quality of the transcriptions. Data were analysed using Python QualCoder version 2.2. We used a framework method for the analysis of data ^30^. Stage 1- *Transcription of the data*- where all interviews conducted in Malayalam were translated into English and transcribed. Interviews conducted in the English language were also transcribed simultaneously. Deidentified transcripts are available as *Underlying data* ^31^. The next stage, stage 2- *Familiarisation with the interviews*- the research team read the interview transcripts multiple times to get familiarised with the data. In stage 3 - *Coding*- the research team coded translated versions of the interviews using inductive coding and generated the initial codes. Coding was completed using Python Qual Coder software. After familiarising the data, the study team read the transcripts line by line. During the open coding procedure, the transcripts were labelled or coded to represent the meaning of each phrase or line. Stage 4- *Developing a working analytic framework*- after coding the first few rich transcripts from different stakeholders and using deductive categories from the interview topic guide, the study team merged the initial similar codes and created overarching categories. The overriding categories thus created were used as the working analytical framework. Stage 5- *Applying the analytical framework*-the working analytical framework was then used for indexing subsequent transcripts using the existing categories and codes. Categories having similar meanings were further merged to form final themes. And finally in stage 6- *Interpreting the data*-the study team analysed the similarities and differences in the data themes among various stakeholders and interpreted them accordingly.

### Positionality of the researcher

The researcher who led the data collection and analysis (SCT) is a trained nurse with a PhD in public health. She is also a trained qualitative researcher. Although her education and training could have an influence on the research, having not experienced the illness or the care seeking experience of the condition that was being researched, she is placed well to do the research and look at it objectively. Being a young female researcher and sharing the same cultural identity as that of the participants, helped her interact with the patients without them being fearful. Nevertheless, being from the same institution and the statements about her association with the research centre may have had some influence on the research participants.

## Results

### Participant characteristics

The study team conducted 22 in-depth interviews with healthcare providers, patients, and caregivers ( Table 1). Among the patients, three were females, five were males, and their ages ranged from 45 to 72 years. The mean age of the HF patients included in the study was 56.3 ± 8.9 years. All three caregivers who participated in the study were females. The cardiologists interviewed in the survey were from private (n=1), co-operative (n=2), and public (n=2) tertiary care hospitals. Out of these centres, the private and public tertiary hospitals had dedicated HF clinics, while the co-operative hospital did not have a dedicated HF clinic. All nurses in the study reported more than one year of experience managing and treating HF patients in intensive care units or HF clinics. All the participants approached for the interview consented to participate in the study.

**Table 1.** List of interviews with patients, caregivers, and health care providers (HCPs).

Serial number	Category	Age/Sex of patients and caregivers and Designation of HCPs	Type of centre used (for patients and caregivers)/ affiliated to (for HCPs)	A dedicated heart failure (HF) clinic is available.
1	Patient 1	60/Female	Public super specialty	Yes
2	Patient 2	49/Male	Public super specialty	Yes
3	Patient 3	45/Male	Public super specialty	Yes
4	Patient 4	63/Male	Public super specialty	Yes
5	Patient 5	63/Male	Co-operative multi-specialty	No
6	Patient 6	58/Male	Public super specialty	Yes
7	Patient 7	72/Female	Private multi-specialty	No
8	Patient 8	50/Female	Private multi-specialty	No
9	Caregiver1	45/Female	Co-operative multi-specialty	No
10	Caregiver2	38/Female	Public super specialty	Yes
11	Caregiver3	48/Female	Private multi-specialty	No
12	Nurse 1	Project nurse	Public super specialty	Yes
13	Nurse 2	Project nurse	Public super specialty	Yes
14	Nurse 3	Project nurse	Private multi-specialty	Yes
15	Nurse 4	Nursing supervisor	Private multi-specialty	Yes
16	Nurse 5	Nursing officer	Co-operative multi-specialty	No
17	Nurse 6	Research Nurse	Private multi-specialty	No
18	Doctor 1	Consultant Cardiologist	Public super specialty	Yes
19	Doctor 2	Consultant Cardiologist	Public super specialty	Yes
20	Doctor 3	Consultant Cardiologist	Private multi-specialty	Yes
21	Doctor 4	Consultant Cardiologist	Co-operative multi-specialty	No
22	Doctor 5	Consultant Cardiologist	Co-operative multi-specialty	No

### Study themes

**Figure 1.**
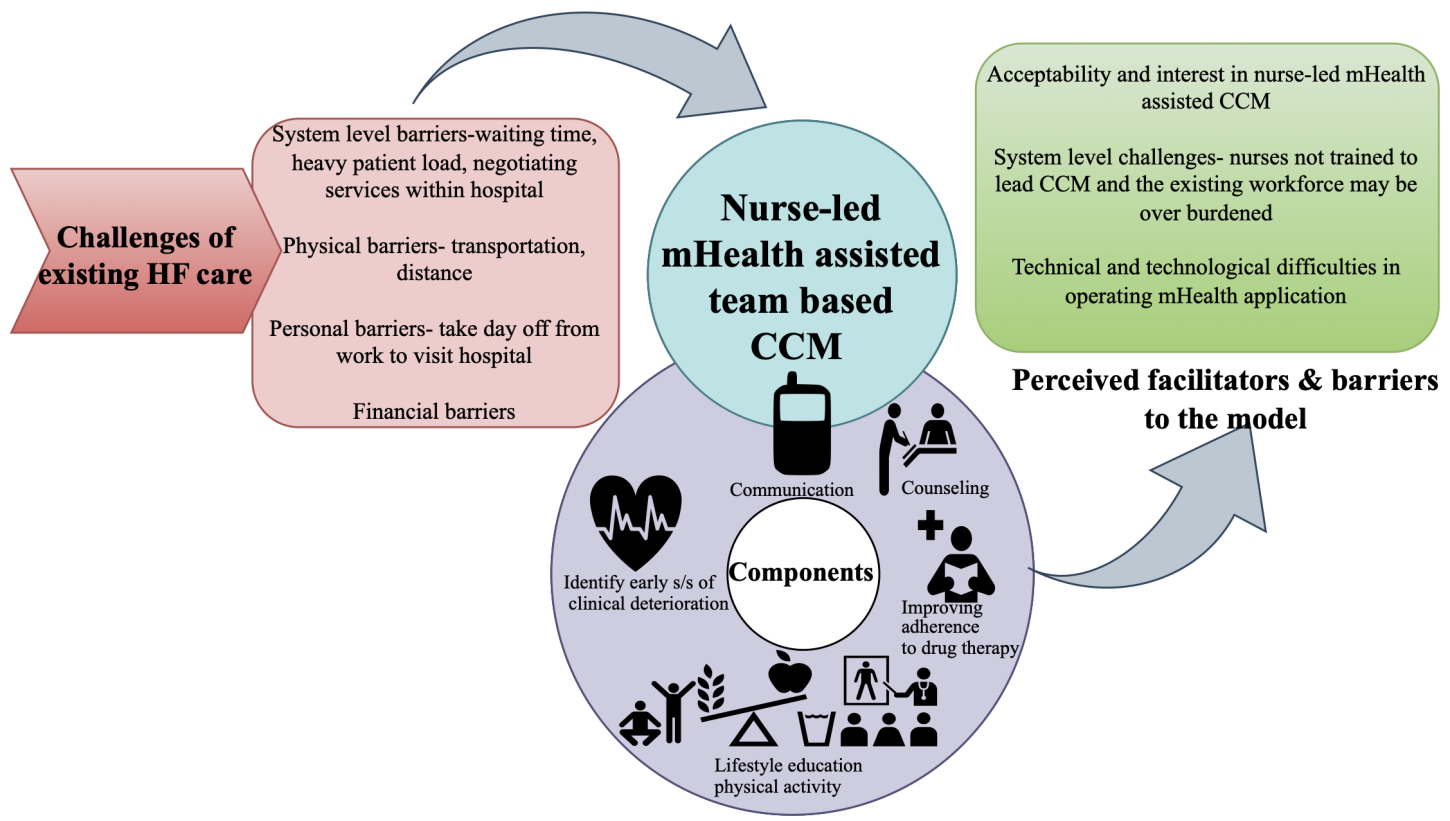
Summary of findings from the multi-stakeholder interviews.

**Figure 2.**
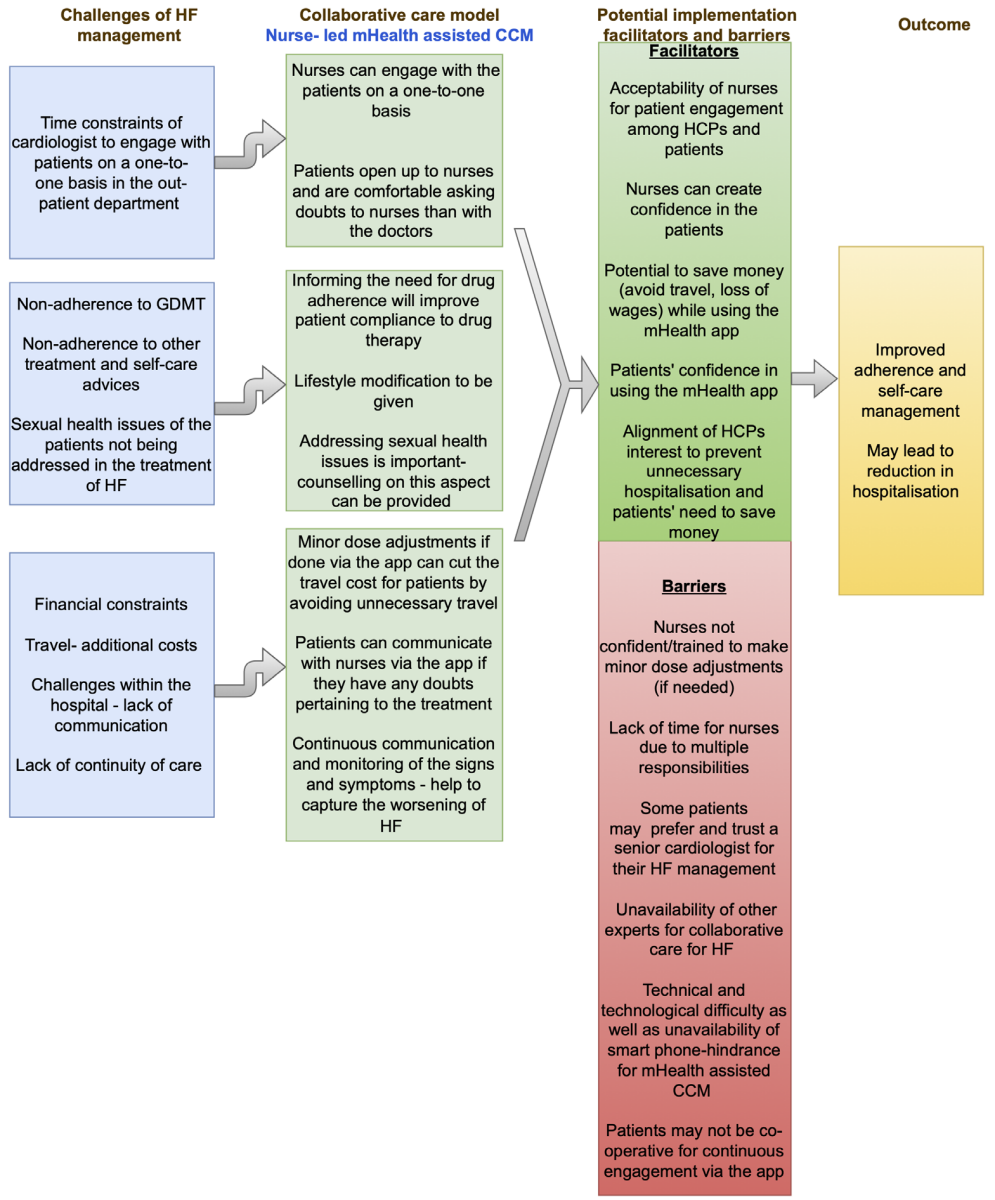
Feasibility of a nurse-led mHealth-assisted CCM for HF management. CCM, collaborative care model; HF, heart failure.

The key findings are presented under three themes: current practices and challenges to HF management, options to tailor the nurse-led and mHealth-assisted model for HF management, and perceived facilitators and barriers of the intervention model. Figure 1 summarises the findings of the multi-stakeholder interview, and Figure 2 gives an overview of the feasibility of a nurse-led mHealth-assisted CCM for HF management. Table 2 presents illustrative quotes to support each theme.

**Table 2.** Themes and illustrative quotes.

Theme	Quotes
Challenges to heart failure (HF) management	“we cannot tell everything to the patient, we get hardly 5 minutes for a patient. In our centre we have 100 out patients daily, so we will not get much time for each patient.” -Doctor 4, Co-operative multi-specialty hospital “Each time mom goes to the hospital, the medicines are to be taken for 7 days, there are medicines to be taken 5 days a week. Even for me it is difficult to remember the medicines. We have doubts whether we took it yesterday or not. Is there any provision of giving the medicines as one strip for a month. Even for youngsters it is difficult to remember, so for mom some days there are five tablets some days there are 4 tablets.” - Caregiver of female patient, Private multi-speciality hospital “They usually do not follow the treatment advices . May be that is the reason for their recurrent admission. They do not follow the intake restrictions and all when they go back home.” - Nurse 5, Co-operative multi-specialty hospital “Nurse: Some tell about financial difficulties Interviewer: so do such patients miss coming for follow-up? Nurse: yeah definitely. some say there are some issues at home or there is financial difficulty, travel is difficult. some medicines are very costly so if they have to take it two times a day that itself is a burden. so they ask if they can continue that tablet itself instead of coming here.” - Nurse-1, Public super specialty hospital “Interviewer: What are their main problems? Is it difficulty in erection due to some medications or something else? Nurse: Yes, difficulty in erection due to medication; that was the problem for a male patient. Not everyone discusses this issue; only one or two ask. Interviewer: So, do they stop medications due to this problem? Nurse: They do not say that they have stopped medicines because of sexual problems; they may not know which treatment may be causing it.” Nurse-3, Private multi- speciality hospital
Collaborative care model	“Patient: Weekly once the nurse calls and ask about health condition, that is very helpful. If I have some problem, I will also call the nurse. Interviewer: In what way is it good? Patient: Good means. When they call and ask if we have some difficulties we can tell them directly or else for that I have to come here (to the hospital). So once I told the nurse that I am having a problem then she asked if there is any hospital nearby. I said yes, so she asked me to show there. When I showed there the doctor told the swelling is due to my disease, so when my disease becomes normal the swelling will subside on its own and told me not to take any medicines. So I continued the medicine from here and 80 percent of my problem is okay now.” - Patient 2, Public super speciality hospital “Involving nurses, it’s a good initiative. It will help the patients to open up about their problems. They used to tell the problems to nurses which they don't open up to the doctors.” -Nurse-3, Private multi- speciality hospital “We can tell, there are two things one is there is a certain group of people, whatever we tell they will not accept, they will just take Lasix and will be happy going on with life. They will not do anything else. So for them we should make them understand what are the benefits of taking the medicines that are prescribed for them. we should take classes for them. we should make them understand the use of each drug, they should feel they have to take it. So with that we can reduce hospital admissions. When our staff tell them they will feel like we should take it. If they take it continuously for a while then the EF will increase and then we can stop the medicines then, so we have to create that confidence in the patients. That is more important.” - Doctor 4, Co-operative multi-specialty hospital “In my opinion, almost every aspect should be covered. Intake restrictions, rehabilitation medications compliance and its adverse effects if not followed.” -Nurse 4, Private multi- speciality hospital “May be patients in that age-group, they need a counselling for sexual health also. But very rarely patients ask these things to us, sometimes they ask. We need counselling in that aspect, we definitely need do not look at it as the patients are inhibited to ask. Sometimes they end up in uncontrolled heart failure because they don't ask and they are not aware. So that should be part of counselling like how much of activity for each patient, tailor made advice should be given.” -Doctor 3, Private multi-specialty hospital “The initial follow-up can be done 2 weeks or 3 weeks then it can be made 3 months or 6 months. But you must monitor sodium and potassium, every month or twice monthly. Dose the nurse can reduce. So, the patient can avoid travel. See the HF patient is travelling 40–50 km and then coming here. Minimum 20 kms, if they are staying near, they can come, but if they are staying far away, we can tell them to join a video call, so that they will be happy.” -Doctor 5, Co-operative multi- speciality hospital
Barriers and facilitators of the model	“This time potassium level was checked and my daughter went to the op and saw doctor. That time doctor said no need to take this these medicines. so we check this and come, and if we come from here to hospital if come in the morning it will be evening when it is done. so this is a good thing. it will be easier when most needed, for instance we test blood in DDRC (lab). the when report comes we can message in the app.” -Patient 4, Public super specialty hospital “In the patient perspective they want the most senior doctor and best doctor to be treating them because it is their heart. Patient will be trusting none other than the cardiologist. There are lot of patients who don't even trust junior cardiologist means they will specifically trust some specific cardiologist only, they want a particular doctor only.” -Doctor 1, Public super specialty hospital “First thing, some of them may not have android mobile. Another thing is the technical difficulty, there should be someone to look into this and enter all these. That is a difficulty, there is time, so we can teach them. Acute admissions they have to come here. But we will know other practical difficulties when we start only.” -Doctor 5, Co-operative multi-specialty hospital “That also will be problem only, because nurse calls them very frequently someone can cut the phone, they may not respond properly, so I don’t know whether they respond every time.” - Doctor 3, Private multi-specialty hospital

### Current practices and challenges to HF management

In study settings with no specialty HF clinic, doctors directly managed and reviewed the patients. Cardiologists often needed more time to engage with each patient but could not do so due to their heavy patient load and other responsibilities within the hospital. Patients attending the outpatient department were often prescribed GDMT and were referred to different departments based on their specific needs.

A senior nurse often assisted the management in hospitals with specialty HF clinics. Nurse-assisted management in specialty HF clinics usually involves more personalised patient engagement. Nurses conducted monthly telephonic reviews with patients and provided advice on dietary and other HF management strategies.

Various challenges to effective HF care delivery were mentioned by healthcare providers, patients, and their caregivers.

***Non-adherence to treatment advice***

Healthcare providers complained about non-adherence to the advice given to their patients. In their opinion, one of the primary reasons for this non-adherence among HF patients was the denial of their diagnosis. Besides medical treatment, patient compliance with self-care recommendations could have been better, as some patients did not follow advice on water intake as suggested and often skipped diuretics. The HCPs also noted a tendency by the patients to use alternate systems of medicine like Ayurveda, which could further complicate the management and adherence to GDMT. In general, HCPs felt that the concurrent use of drugs from other systems of medicine resulted in poor compliance with GDMTs.

*“There are patients who stop medication on their own or by hearing the opinion of others. There are so many other patients who follow Ayurvedic treatment after hearing others’ opinions; even after giving so much advice, they used to seek other treatment modalities in our place. For example, recently, a patient stopped our treatment and followed Ayurvedic treatment. His symptoms worsened, and the patient recently underwent coronary artery bypass grafting (CABG).”*
  -     Nurse-3, Private multi- speciality hospital

Non-adherence to GDMT was perceived to be more common among male patients, possibly due to concerns about side effects causing erectile dysfunction, as noted by the nurses.

*“Nurse: We don’t get enough time to call them and ask. Patients complains about the non-adherence to medication. Usually they don’t open up about it promptly but through communication we find such problems (sexual health related) in non-adherence to medication.*
*Interviewer: What is the basic reason for not taking the medications among the patients? Is it financial difficulties?*
*Nurse: Usually the male patients have the non-compliance of the medications. They tell me they have erection problems.”*
  -     Nurse-4, Private multi- speciality hospital

Another challenge mentioned by a caregiver, particularly for older people, was their difficulty recalling all the instructions and medicines they needed to take. They believed that constant reminders or direct administration of medications by a caregiver were necessary to ensure adherence.

While the HCPs observed non-adherence to treatment advice, the interviewed patients reported they followed it diligently. According to one of the patients, adhering to the treatment plan and regular follow-up as advised by the doctor effectively managed their condition. Patients reported being aware of the necessary precautions and strictly followed dietary recommendations and water intake instructions.

***Continuity of care***

According to the nurses, patients do not come for follow-up as advised, resulting in high mortality among patients with HF. Financial constraints, including travel expenses, hindered patients from attending follow-up appointments. Nurses also mentioned that in some instances, patients with no symptoms often skipped follow-ups, and some missed them due to difficulties in understanding the instructions. For example, when advised to return for a follow-up in three or six months, some patients perceive that frequent follow-ups are not necessary for HF patients. It leads to missed follow-ups and subsequent medication discontinuation.

Language barriers in communicating with doctors, brief interaction with doctors, multiple doctors attending to the patient within the hospital, and technical jargon were perceived as challenges to continuity of care. Even though language barriers, multiple doctors attending to the patients were specific to patients seeking care from one of the tertiary super-specialty hospital, where the treating doctors are from different parts of the country, these experiences are very important from the patient perspective in their continuity of care. Patients also faced difficulty reaching doctors for dose adjustment based on their laboratory results.

Patients highlighted financial constraints as a major barrier to accessing treatment. Many patients could not continue working after being diagnosed with HF, which has seriously limited their source of income. To save medication costs, some relied on Karunya pharmacy, a government-subsidised pharmacy in Kerala offering medicines at a lower price than public sector pharmacies. However, patients noted that Karunya pharmacy did not stock all the prescribed drugs.

### Options to tailor the nurse-led and mHealth-assisted model for HF management

HCPs believed that continuous monitoring through a mHealth application may help to capture worsening HF. Cardiologists felt a nurse-led CCM for HF could increase treatment and self-care adherence. Given their time limitations to engage with patients in detail during outpatient visits, HCPs appreciated the role of nurses in managing HF.

Cardiologists and nurses suggested different CCM components;

***Regular monitoring of blood parameters***

Relevant blood parameters for each patient should be monitored every 2–3 weeks, and the medicine dose can be titrated based on that. Nurses who have undergone training can adjust the dose with the doctor’s approval. This can be conveniently managed through the mHealth application, sparing patients from unnecessary hospital visits.

*“But you have to monitor sodium and potassium every month or twice monthly. Nurses (can reduce the dose. So the patient can avoid travel. See, the HF patient is traveling 40-50 km and coming here; if they are staying near, they can come and show, but if they are staying far away, we can tell that there is no problem if we keep a video call also they will be happy.”*
  -     Doctor 5, Co-operative multi- speciality hospital

***Lifestyle modification classes***

HCPs advised providing diet and physical activity classes, focusing on graded activities for young HF patients. HCPs suggested prioritising the physical activity component and alleviating patients' fear of engaging in physical activity. They also recommended including awareness sessions on alcohol and smoking cessation. HCPs emphasized the importance of good rest and sleep in the intervention since these aspects cannot be covered in detail in regular outpatient settings. Therefore, sharing these tasks with a dedicated nurse may help to improve adherence to self-care strategies. Additionally, adapting the activity or diet to the patient’s concurrent chronic conditions was recommended.

***Counselling support for HF patients and their caregivers***

HCPs recognised the potential need for counselling support for patients’ sexual health concerns. They noted that some patients on certain HF drugs may have sexual health issues, and the sexual health needs of patients and their partners often lead to significant distress in families. Nurses reported that female patients expressed anxiety about having sexual relations with their spouses, while male patients mentioned difficulties with erections, primarily those under 50 years old. HCPs noted that many patients refrained from engaging in sexual activity due to fear, resulting in unmet sexual needs.

Additionally, HCPs highlighted that patients often hesitate to discuss their sexual health concerns with doctors, especially when family members other than their wives are present. However, some of the nurses in the study noted that patients felt more comfortable discussing these issues with nurses.

*“Some patients used to ask me about sexual problems, but most of them did not ask about it to the doctor. It affects their mental health. As it is a serious issue, we used to tell them to discuss it with the doctor and get medicines if needed. Recently, one of the patients asked about this problem, although they were stressed to ask me. There was also a female patient who asked these questions.”*
  -     Nurse-3, Private multi- speciality hospital

HCPs interviewed recognised the importance of involving family members in caring for HF patients to ensure continuity of care. They emphasised the significance of caregivers, especially for older patients. They believed that even when family support is limited, proactive efforts by HCPs to engage the family in care could benefit the patients. Furthermore, HCPs recommended recognising the caregiver burden and extending psychological support to caregivers in the CCM.

*“We have to consider the psychosocial support for caregivers. Like any other chronic illness, caregivers have much stress. They should be given support because sometimes they will have financial problems. So, there should be some psychosocial support. As part of CCM, we should not focus on the patient alone.”*
  -     Doctor 3, Private multi-specialty hospital

***Constant communication with patients***

Cardiologists felt that nurses could actively engage with patients and educate them about the importance of drug adherence in preventing exacerbations. HCPs were optimistic that emphasising the importance of medication adherence would eventually enhance patient compliance and reduce hospital admissions.

HCPs recognised the role of the mHealth application for continuous patient monitoring. They believed that real-time monitoring through mHealth could effectively detect worsening HF symptoms, enabling prompt intervention to prevent adverse outcomes. Nurses saw potential in the mHealth application for sending reminders to patients about follow-up appointments.

*“Yes, the app will be helpful because we measure the patient's heart rate, respiration, edema, everything. If there are any changes, we can adjust the dose immediately.”*
  -        Doctor 4, Co-operative multi-specialty hospital

### Perceived facilitators and barriers of the intervention model

HCPs were optimistic about the nurse-led mHealth-aided CCM for HF management. They felt that nurses could instil confidence in patients in self-management and improve treatment adherence. Both HCPs and patients viewed nurses as an excellent choice to lead CCM. According to the nurses, patients openly discussed their concerns with nurses without hesitation. Patients were happy to engage with nurses in managing their health conditions.

Cardiologists were optimistic about introducing minor dose adjustments through the mHealth mobile application. Both cardiologists and nurses saw the potential benefit of the mHealth tool for patients, as it reduces the need for hospital visit to modify their dosage. However, they felt that the feasibility of the mHealth app-based interventions could depend on the nurses' patient load. HCPs believed that convincing patients about the benefits of mHealth, such as avoiding unnecessary hospitalisation and saving money on health care, could increase patient adoption of mHealth applications.

*“See, the main issue is money. To buy medicines, they need money; Some medicines are very costly. They are not thinking about how they can reduce the medicines. So, I think it will work. They must come here for follow-up and wait here. Sometimes, they may not get a vehicle. There won’t be anyone to accompany them. Children will be staying away, and they must take leave. So, I think, in that sense, the mHealth app will work. They do not have to take leave. Every week, they get a prescription. Sometimes they may have to do echo and, in such instances, maybe app alone will not work.”*
  -     Nurse-2, Public super specialty hospital

Patients identified several facilitators that made them receptive to nurse-led mHealth intervention. They recognised the potential of the mHealth application in avoiding unnecessary hospital visits. Many patients believed they required dose adjustments for their medicines and felt the mHealth application might facilitate this without hospital visits. Patients expressed confidence in using the application with appropriate guidance from nurses. They also added that promoting home-based monitoring of blood pressure, weight, and blood glucose would facilitate increased engagement with the mHealth application for updating these details and enable them to receive prompt management support.

While the nurse-led CCM received positive feedback from HCPs, some implementation challenges were highlighted. Cardiologists suggested training nurses on the basics of HF medications, their side effects, and contraindications. Nurses expressed their need for more confidence in medication dose adjustments to manage HF. Additionally, a few doctors observed that nurses might hesitate to make treatment decisions due to a lack of confidence. The complexity of the patient’s cardiac condition, potential drug interactions, and the nurse’s inexperience in tapering medications were reasons for the nurse’s reluctance to make treatment decisions.

All HCPs observed that doctors and nurses had to juggle HF clinic activities, regular ward duty, and telephonic follow-ups. They recommended having a full-time nurse to manage CCM-related activities. Further, nurses stressed the importance of cooperation and support from respective doctors for the effective implementation of the model.

Cardiologists appreciated the involvement of physiotherapists, dieticians, psychologists, urologists, and diabetologists in the CCM. However, they emphasised these specialists' unavailability and hectic work schedules for HF management. Doctors suggested that the nurses could be trained in CCM to provide physiotherapy and counselling to HF patients. However, cardiologists recommend specialised care for patients with serious illnesses.

All stakeholders identified technical difficulties and technology-related unawareness as significant challenges in delivering mHealth interventions. Additionally, they acknowledged the barriers posed by the unavailability of smartphones and internet access. HCPs expressed concern about the patient's comprehension level, as some patients might not follow application-related instructions correctly. Further, age and education level could affect a patient’s ability to use mobile phones effectively since some older patients may face difficulties in viewing and typing messages using a phone. HCPs emphasised that owning a smartphone did not necessarily imply proficiency in using it.

A nurse mentioned that patients might be concerned about frequent phone calls from the hospital, viewing them as disturbances. She felt that some patients might become annoyed when they receive regular calls and may be hesitant to disclose information. However, the nurses emphasised the importance of ongoing nurse-patient telephonic conversations to build patient acceptance and trust.

## Discussion

Our findings highlight the challenges in HF management and the feasibility of a nurse-led mHealth-assisted CCM for HF. Barrier and facilitators in implementing the team based collaborative care model, facilitated by mHealth enabled and trained nurses, for management of heart failure (TIME-HF) were highlighted which helped to modify the model.

System-level challenges, high patient load limiting cardiologists from providing dedicated outpatient care, and factors like multiple consultations, long waiting times, language barriers in communicating with patients, and patient non-compliance resulted in a lack of follow-up and continuity of care for HF patients. Financial constraints, as well as indirect costs due to travel, were also rendered as hindrances in accessing care. Various barriers like financial, transportation, and difficulties in navigating the healthcare system were reported to limit patient’s access to quality care ^32^.

Doctors and nurses noted non-adherence to treatment advice among HF patients, often due to denial and fear of side effects. Some turned to alternate medicines following lay advice. Other studies that reported alternate medicine following secular direction were noted to have contributed to poor adherence, one of the major challenges in HF management ^33– 35^. Even though the HCPs reported poor adherence, patients claimed strict adherence to doctors’ advice. One reason for this could be because we have selected patients who had come to the hospital for their follow-up. Studies have reported a low medication adherence among patients with HF and impact of non-adherence on HF outcomes ^36– 40^.

Recognising various barriers to HF management like system-level barriers, long waiting time, inability to access care, multiple consultations, physical barriers, distance and transportation, personal barriers, denial, fear of side-effects, and financial barriers, HCPs favoured a nurse-led mHealth assisted CCM for providing optimal HF care. Nurses were considered ideal for leading the CCM. Cardiologists and patients acknowledged their effectiveness and acceptability in imparting patient confidence for self-management. Patients expressed greater comfort in discussing their needs with nurses than doctors, perceiving nurses as key care coordinators due to their proximity to individuals requiring care, as seen in other studies ^41^.

HCPs emphasised that CCM should focus on creating awareness of treatment/drug adherence to reduce unnecessary and frequent hospitalisation. Caregiver accounts highlight the challenges older adults face in adhering to medication, underscoring the family’s vital role in treatment compliance. Given the complex HF treatment regime and associated self-care, especially for older adults, family support is essential ^42^. It leads to reduced hospital readmissions and improved medication adherence ^43^. Caregivers play a crucial role in HF management, but caregiver burden is often overlooked. Caregivers expressed their need to receive psychological support and be part of the counselling sessions.

Patients experience considerable distress due to unmet sexual needs. While patients hesitate to discuss sexual health matters with their doctors in a busy outpatient clinic, some have confided in nurses. Evidence suggests that only a few HF patients receive sexual health counselling ^44^. In a study aimed at assessing HF patients’ informational needs regarding sexual health, patients required information related to handling HF symptoms during sexual activity, insights into maintaining relationships, and knowledge of relaxation techniques ^45^.

Despite the general support for the mHealth-assisted CCM, some stakeholders voiced concerns about its feasibility in low-resource settings. Nurses may require enhanced training and confidence to lead the decision-making, particularly in adjusting medication doses. Given the constraints, HCPs recommended a dedicated training session on the pharmacological management of HF for nurses as part of the CCM. Nurses considered the cooperation of patients and doctors as intrinsic for the mHealth application-based intervention to be successful. In addition, while the mHealth application could benefit elderly patients, age can be a significant barrier to handling the phone ^46^. Among older people, the educated group may be better off than their less educated counterparts in using the phone. Age and education are also significant barriers, as noted by the HCPs. While patients felt the mHealth application-based intervention would significantly cut down their travel to the hospital, especially for minor dose adjustments, their concern centred on the availability of a phone that supports the mHealth application and their ability to use such devices. The nurses raised affordability and privacy issues as additional barriers to implementing mHealth as part of HF care.

Although there were challenges to overcome, favourable factors that could facilitate the intervention implementation outnumbered the barriers. Patients appreciated the potential cost-saving related to travel and missed workdays, which could make using the mHealth-based intervention more acceptable. Their confidence in managing the mHealth application suggests their receptiveness to this type of intervention. Additionally, healthcare professionals are interested in reducing unnecessary hospitalisations and helping patients save money, which could also facilitate the adoption of the intervention.

## Adapting the CCM model in TIME-HF

We recruited dedicated nurses who are trained in CCM for HF management. These nurses underwent comprehensive training on various aspects of HF management, including medical management, behavioural counselling, physical activity, and sexual health education for patients as part of the study. We encouraged the selected nurses to engage patients in discussions about their sexual health needs. The training component, handbooks for nurses, and the decision support system in the mHealth application were improvised to improve the confidence of nurses engaged in the TIME-HF study. Additionally, we developed a handbook in the local language that covers all aspects of HF, incorporating feedback from healthcare professionals. It served as a quick reference for patients to understand the technical aspects of HF, medication actions, and potential side effects. We have also improvised the mHealth application and included voice and image-based communication for better acceptability among patients.

## Conclusions

Our research revealed several obstacles in the management of HF in Kerala. These include inadequate patient-centred care, communication difficulties between cardiologists and patients in the outpatient setting, challenges in real-time monitoring of warning signs and symptoms, lack of follow-up and continuity of care, financial constraint including travel expenses, inadequate attention to sexual health and other psychological needs of patients, and apprehension regarding physical activity. A collaborative care model led by nurses, which could assist in addressing some of these challenges, has been widely accepted by multiple stakeholders, including patients and their caregivers. Healthcare professionals were optimistic about the CCM but insisted on nurses receiving adequate training. Mobile health (mHealth) application was seen as a promising tool to assist nurses in managing HF and could benefit patients by reducing the need for unnecessary travel. Effective implementation of CCM may lead to reduced hospitalisations and improve patients' ability to manage their HF symptoms.

### Limitations

We had selected only those patients who had come to the hospitals for follow-up. We noted that when HCPs had stated non-adherence to treatment by patients, patients were not reporting the same. It could be because we had selected patients who came to the hospital for follow-up and therefore, in terms of general compliance, we were seeing patients who were already compliant in terms of visiting their doctors. We might have got patients who might have been generally compliant. Additionally, patients could also perceive the researchers as part of the same hospital, causing social desirability bias, which could influence their response on treatment adherence.

## Data Availability

Figshare: Interview transcripts. https://doi.org/10.6084/m9.figshare.25303423 ^31^ The file “Interview transcripts” contains the following underlying data: •   Interview transcripts of patients (deidentified) •   Interview transcripts of caregivers (deidentified) •   Interview transcripts of doctors (deidentified) •   Interview transcripts of nurses (deidentified) Figshare: In-depth Interview guides. https://doi.org/10.6084/m9.figshare.25303396 ^28^ The file “In-depth Interview guides” contains the following extended data: •   In-depth interview guides used for patients, caregivers, doctors and nurses. Figshare: COREQ checklist for “Feasibility of a nurse-led, mHealth-assisted, and team-based collaborative care model for heart failure care in India: Findings from a multi-stakeholder qualitative study.” https://doi.org/10.6084/m9.figshare.25303387 ^29^ Data are available under the terms of the Creative Commons Attribution 4.0 International license (CC-BY 4.0).
