# Supplementary material for: Feasibility of a nurse-led, mHealth-assisted, and team-based collaborative care model for heart failure care in India: Findings from a multi-stakeholder qualitative study
Source: Wellcome Open Res. 2024 Oct 15;9:219. Originally published 2024 Apr 24. [Version 2] doi: 10.12688/wellcomeopenres.21175.2 (PMC11358683; doi:10.12688/wellcomeopenres.21175.2)
Supplement: Peer review history

# Reviewer report: Reviewer response for version 2

**DOI:** 10.21956/wellcomeopenres.25592.r105101

**Published:** 2024-11-05

**Authors:** GK Mini

**Affiliations:** 1 Global Institute of Public Health, Ananthapuri Hospitals and Research Institute (Ringgold ID: 75682), Trivandrum, Kerala, India

No further comments

Is the work clearly and accurately presented and does it cite the current literature?

Yes

If applicable, is the statistical analysis and its interpretation appropriate?

Partly

Are all the source data underlying the results available to ensure full reproducibility?

Yes

Is the study design appropriate and is the work technically sound?

Yes

Are the conclusions drawn adequately supported by the results?

Yes

Are sufficient details of methods and analysis provided to allow replication by others?

Partly

Reviewer Expertise:

Public Health, Biostatistics

I confirm that I have read this submission and believe that I have an appropriate level of expertise to confirm that it is of an acceptable scientific standard.

# Reviewer report: Reviewer response for version 2

**DOI:** 10.21956/wellcomeopenres.25592.r105100

**Published:** 2024-10-24

**Authors:** Alan Crouch, Lyndell Manson

**Affiliations:** 1 The University of Melbourne, Melbourne, Victoria, Australia; 2 Arts and Humanities, The University of Melbourne (Ringgold ID: 2281), Melbourne, Victoria, Australia

However, I have reviewed the authors response to Lyndell Manson's and my comments and am now satisfied that these address the key issues raised.

Is the work clearly and accurately presented and does it cite the current literature?

Yes

If applicable, is the statistical analysis and its interpretation appropriate?

Not applicable

Are all the source data underlying the results available to ensure full reproducibility?

Yes

Is the study design appropriate and is the work technically sound?

Partly

Are the conclusions drawn adequately supported by the results?

Partly

Are sufficient details of methods and analysis provided to allow replication by others?

Partly

Reviewer Expertise:

NA

We confirm that we have read this submission and believe that we have an appropriate level of expertise to confirm that it is of an acceptable scientific standard.

# Reviewer report: Reviewer response for version 1

**DOI:** 10.21956/wellcomeopenres.23420.r92870

**Published:** 2024-08-28

**Authors:** Christi Deaton

**Affiliations:** 1 University of Cambridge, Cambridge, UK

The paper is clearly written and addresses an important topic: HF management in LMIC. References could be updated: (2012 guidelines), reference 1st sentence not relevant (epidemiology HFpEF not HF QoL), little review of nurse-led mgmt. Sample size small across 3 groups (8 pts - text needs correction as there are 5 male pts not 4) for robust conclusions although from 5 hospitals. Analysis done well, and findings clearly presented illustrating patient challenges and need for nurse training and better patient support. It seems to have informed TIME-HF study, but not discussed until end.  Paper would be stronger if relation to TIME-HF discussed earlier and how findings from this analysis influenced TIME-HF (place this analysis in context). Reference needed for TIME-HF (doi:10.12688/wellcomeopenres.19196.2.)

Is the work clearly and accurately presented and does it cite the current literature?

Partly

If applicable, is the statistical analysis and its interpretation appropriate?

Not applicable

Are all the source data underlying the results available to ensure full reproducibility?

Yes

Is the study design appropriate and is the work technically sound?

Partly

Are the conclusions drawn adequately supported by the results?

Yes

Are sufficient details of methods and analysis provided to allow replication by others?

Yes

Reviewer Expertise:

diagnosis and management of patients with HFpEF; cardiovascular prevention; nurse and AHP led interventions.

I confirm that I have read this submission and believe that I have an appropriate level of expertise to confirm that it is of an acceptable scientific standard.

# Response

**Published:** 2024-10-10

**Authors:** Panniyammakal Jeemon

**Affiliations:** Sree Chitra Tirunal Institute for Medical Sciences and Technology, Trivandrum, India

Dear Sir/Madam, Thank you for your thoughtful comments. Please find below the detailed response to the comments. We hope they satisfactorily address all the points raised. **1. The paper is clearly written and addresses an important topic: HF management in LMIC. References could be updated: (2012 guidelines).** Thank you for pointing this out. We have updated the reference. McDonagh TA, Metra M, Adamo M, et al.: 2021 ESC Guidelines for the diagnosis and treatment of acute and chronic heart failure. the task force for the diagnosis and treatment of acute and chronic heart failure 2012 of the European Society of Cardiology. Developed in collaboration with the Heart Failure Association (HFA) of the ESC.  Eur Heart J. 2021;42(36):3599–726. doi:10.1093/eurheartj/ehab368 **2. Reference 1st sentence not relevant (epidemiology HFpEF not HF QoL)** Thank you. We have changed it. **3. Little review of nurse-led mgmt** Thank you we have added relevant literature on nurse-led management Studies have shown that nurse-led management in HF with regular follow-up and patient education has positive impact on patients with improved compliance, and quality of life as well as increased survival and decreased number of hospitalizations (14–17). The references are as follows:  Wu X, Li Z, Tian Q, Ji S, Zhang C: Effectiveness of nurse-led heart failure clinic: A systematic review. International Journal of Nursing Sciences. 2024;11(3):315–29. 10.1016/j.ijnss.2024.04.001 Stromberg A. Nurse-led heart failure clinics improve survival and self-care behaviour in patients with heart failure Results from a prospective, randomised trial. Eur Heart J. 2003 ;24(11):1014–23. Rich MW, Beckham V, Wittenberg C, et al.: A multidisciplinary intervention to prevent the readmission of elderly patients with congestive heart failure. N Engl J Med. 1995;333(18):1190–5. 10.1056/NEJM199511023331806 Jaarsma T: Effects of education and support on self-care and resource utilization in patients with heart failure. Eur Heart J. 1999;20(9):673–82. **4. Sample size small across 3 groups (8 pts - text needs correction as there are 5 male pts not 4) for robust conclusions although from 5 hospitals** Thank you. We have corrected it in the article. **5. Analysis done well, and findings clearly presented illustrating patient challenges and need for nurse training and better patient support. It seems to have informed TIME-HF study, but not discussed until end. Paper would be stronger if relation to TIME-HF discussed earlier and how findings from this analysis influenced TIME-HF (place this analysis in context).** We have added in the introduction part the need for this study as well in the beginning of the discussion. Therefore, this study aimed to obtain perceptions of potential challenges to heart failure care and suggestions on multiple components of the proposed CCM in managing HF and its feasibility. Since this model envisaged a nurse-facilitated CCM, we also were looking for the acceptability of nurses in leading this collaborative care. Additionally, we sought to obtain feedback from stakeholders regarding various aspects of the mHealth model. The findings from the study can help tailor the team-based CCM model (TIME-HF) to adapt it to the cultural context. **6. Reference needed for TIME-HF (doi:10.12688/wellcomeopenres.19196.2.) ** Thank you. We have added the reference. Ref 18- Jeemon P, Bahuleyan CG, Chandgalu Javaregowda D, et al.: Team based collaborative care model, facilitated by mHealth enabled and trained nurses, for management of heart failure with reduced ejection fraction in India (TIME-HF): design and rationale of a parallel group, open label, multi-centric cluster Randomised Controlled Trial [version 2; peer review: 2 approved]. Wellcome Open Res. 2023;8:197. 37795133 10.12688/wellcomeopenres.19196.2 10545985 

# Reviewer report: Reviewer response for version 1

**DOI:** 10.21956/wellcomeopenres.23420.r92875

**Published:** 2024-08-27

**Authors:** Alan Crouch, Lyndell Manson

**Affiliations:** 1 Arts and Humanities, The University of Melbourne (Ringgold ID: 2281), Melbourne, Victoria, Australia; 2 The University of Melbourne, Melbourne, Victoria, Australia

**Summary of paper:**

**Feasibility  of a nurse-led, mHealth-assisted, and team-based collaborative care model for heart failure care in India: Findings from a multi- stakeholder qualitative study**

 Using both inductive and deductive thematic analytical techniques applied to transcriptions of English translations of audio-recorded local language semi-structured stakeholder interviews, the authors of this paper have sought to investigate the feasibility of supported nurse-led models for improvements in the care and management of people living with heart failure. Study findings comprised attributed thematic descriptors across a range of issues including continuity of care, disease parameter monitoring, patient behavioural and lifestyle factors, and service provider - patient communications. Discussion focused on stakeholder perceptions of facilitators and barriers to the introduction of the nurse-led model and the study conclusions highlighted the obstacles to management of heart failure in the setting, while promoting the reported general acceptability and potential utility of the nurse-led collaborative care model.

**General Comments**

 The paper is a generally well written description of a qualitative health system study in my opinion. However, there are epistemological and methodological issues requiring attention.

- In terms of the paper title and its relation to the paper contents, and consistent with Reviewer 1 comments, some additional description in the body text of the nature of the collaborative care model is needed, including clear definition of the nurse role in relation to other clinical stakeholders, patients and their carers,  the integration of mHealth applications and the broad mechanisms by which this collaboration might be  managed; also, how descriptions of this mechanism might be integrated into Figure 2.
- For readers unfamiliar with the country setting, it would be useful to expand on the particular mHealth apps referenced in the paper and how equitable access across the socio-economic spectrum to these apps (and the technology needed to support them) might influence recommendations for further research.
- Inclusion of more peer reviewed reports of recent developments in telephony and smart phone ‘Patient Reported Outcome’ applications into the reference list and discussion of their potential for use as part of the mHealth suite of applications  in the ‘collaborative care model’ might be worthy of consideration by the authors

**Specific Comments:**

**Methodology**

- While the thematic analysis descriptions are comprehensive, it would be useful to reference these techniques, especially for clinical practitioner readers who may not be familiar with this qualitative approach.
- I have some reservations about the description of the inductive thematic analysis and the use of author-generated, unreferenced interview topic guides and questions as the source of the codes for the deductive analysis of the interview transcripts, as follows:
   
  - Analysis of texts is typically conducted until inductive thematic saturation has ‘plateaued’. The description of the process in the methodology section (page 5 under ‘Data Analysis’) needs to indicate that saturation of inductively attributed themes was observed and how this then relates to the ‘the first few rich transcripts’ as reported.
  - Deductive analysis typically requires the application of a ‘specific’ theoretical framework to data, enabling the investigation of concepts within a well-defined context.
  - As you are using the interview topic guides as your ‘theoretical framework’, could you indicate in the paper’s methodology the role of the relevant literature (i.e. from your literature review: on nurse-led out-patient coronary care; team-based collaborative coronary care models; mHealth type telephony applications etc.) in the development of the questions and prompts. The paper needs a defence of the use of the author-generated, currently unreferenced interview topic guides as your ‘theoretical framework’ for deductive analysis.
  - If this is not possible, could you include in a Limitations section of the paper the potential for confirmation / researcher bias in the findings and their interpretation?
  - Given the use of the interview topic guide as themes for deductive analysis, without knowledge of the peer-reviewed evidence underlying the development of questions (and prompts) for the respective stakeholder categories, the issue of confirmation / researcher bias in the paper cannot be assessed. This link to the article “Issues of validity and reliability in qualitative research” may be of use in responding to these comments: https://ebn.bmj.com/content/18/2/34
- The assessment of the integrity of qualitative research requires evidence in the paper of the researchers’ awareness of potential (generally unconscious) bias in the research design and its analysis and reporting. I have included a link to the relevant JBI International Critical Appraisal Tool for Qualitative Research download page: https://jbi.global/critical-appraisal-tools - Could you review the overall checklist, with special attention to questions 6 and 7 (copied below) in the list and include relevant responses in your text?

6.           Is there a statement locating the researcher culturally or theoretically?

 7.           Is the influence of the researcher on the research, and vice- versa, addressed?

**Discussion and Conclusions**

 The paper has reported that medication adherence was an issue from clinician perspectives but noted that this was not a theme in patient transcripts. Given the disproportionately small sample size of patients (8 of 22 participants) in the study, further evidence (e.g. from peer-reviewed studies on the subject) is required to support the conclusion. Further, as no patient reported non-adherence to medication, barriers to treatment compliance from the patient perspective could not be identified. It is recommended that a separate Limitations section be added to the paper and that this issue and others mentioned above be included under this heading. A recommendation for further research on patient perspectives of treatment non-adherence is also indicated.

 In order that the congruence between Findings and the Discussion and Conclusion elements of the paper can be affirmed, the epistemological issues related to the theoretical location of the researchers in the qualitative study and reflections on their influence on the design and execution of the study is required. Methodologically, the rigor of a literature-informed theoretical framework required for the deductive aspect of the thematic analysis should be addressed. Otherwise, the questions of confirmation / researcher bias remain.

Is the work clearly and accurately presented and does it cite the current literature?

Yes

If applicable, is the statistical analysis and its interpretation appropriate?

Not applicable

Are all the source data underlying the results available to ensure full reproducibility?

Yes

Is the study design appropriate and is the work technically sound?

Partly

Are the conclusions drawn adequately supported by the results?

Partly

Are sufficient details of methods and analysis provided to allow replication by others?

Partly

Reviewer Expertise:

Population Health

We confirm that we have read this submission and believe that we have an appropriate level of expertise to confirm that it is of an acceptable scientific standard, however we have significant reservations, as outlined above.

# Response

**Published:** 2024-10-10

**Authors:** Panniyammakal Jeemon

**Affiliations:** Sree Chitra Tirunal Institute for Medical Sciences and Technology, Trivandrum, India

Dear Sir/Madam,

 Thank you very much for sharing your thoughtful comments. We greatly appreciate the comments on our manuscript, and we feel that they have helped us to further strengthen the manuscript. Please find below our detailed response to your comments. 

**1. In terms of the paper title and its relation to the paper contents, and consistent with Reviewer 1 comments, some additional description in the body text of the nature of the collaborative care model is needed, including clear definition of the nurse role in relation to other clinical stakeholders, patients and their carers, the integration of mHealth applications and the broad mechanisms by which this collaboration might be managed; also, how descriptions of this mechanism might be integrated into Figure 2.**

 Thank you for the comments, we have added it in the article.  We are developing a team-based collaborative care model (CCM), facilitated by trained nurses, for managing HF in India. We conceptualized the team-based collaborative care model as a team-based approach to HF care with nurses as a key team player (Panniyammakal, J et al 2023). The reference to the protocol paper that elaborates on the CCM for HF is added as a reference. Ref 18- Jeemon P, Bahuleyan CG, Chandgalu Javaregowda D, et al.: Team based collaborative care model, facilitated by mHealth enabled and trained nurses, for management of heart failure with reduced ejection fraction in India (TIME-HF): design and rationale of a parallel group, open label, multi-centric cluster Randomised Controlled Trial [version 2; peer review: 2 approved]. Wellcome Open Res. 2023;8:197. 37795133 10.12688/wellcomeopenres.19196.2 10545985

**2. For readers unfamiliar with the country setting, it would be useful to expand on the particular mHealth apps referenced in the paper and how equitable access across the socio-economic spectrum to these apps (and the technology needed to support them) might influence recommendations for further research.**

 Thank you. We have explained the mhealth app in the protocol paper. We are adding it as a reference here. In the CCM, we have a mHealth component with two linked applications one for patients and another for nurses, which is developed exclusively for the intervention. The details of the mHealth intervention are explained in the protocol paper. The mHealth patient application is available in Google Play. (Panniyammakal, J et al 2023). Ref 18- Jeemon P, Bahuleyan CG, Chandgalu Javaregowda D, et al.: Team based collaborative care model, facilitated by mHealth enabled and trained nurses, for management of heart failure with reduced ejection fraction in India (TIME-HF): design and rationale of a parallel group, open label, multi-centric cluster Randomised Controlled Trial [version 2; peer review: 2 approved]. Wellcome Open Res. 2023;8:197. 37795133 10.12688/wellcomeopenres.19196.2 10545985

**3. Inclusion of more peer-reviewed reports of recent developments in telephony and smartphone ‘Patient Reported Outcome’ applications into the reference list and discussion of their potential for use as part of the mHealth suite of applications in the ‘collaborative care model’ might be worthy of consideration by the authors**

 Thank you for the suggestion. We have added it.  Mobile applications have reportedly increased the efficiency of monitoring patient-reported outcomes, especially in patients with chronic conditions including cardiovascular diseases, who require continuous monitoring by healthcare providers. It has also been reported that these apps can help patients with chronic conditions to have greater improvements in their satisfaction levels to decrease complications and fewer hospital admissions.

**4. While the thematic analysis descriptions are comprehensive, it would be useful to reference these techniques, especially for clinical practitioner readers who may not be familiar with this qualitative approach.**

 Thank you we have added the reference. Gale NK, Heath G, Cameron E, et al.: Using the framework method for the analysis of qualitative data in multi-disciplinary health research. BMC Med Res Methodol. 2013;13(1):117. 10.1186/1471-2288-13-117

**5. Analysis of texts is typically conducted until inductive thematic saturation has ‘plateaued’. The description of the process in the methodology section (page 5 under ‘Data Analysis’) needs to indicate that saturation of inductively attributed themes was observed and how this then relates to the ‘the first few rich transcripts’ as reported.** **○ Deductive analysis typically requires the application of a ‘specific’ theoretical framework to data, enabling the investigation of concepts within a well-defined context.** **○ As you are using the interview topic guides as your ‘theoretical framework’, could you indicate in the paper’s methodology the role of the relevant literature (i.e. from your literature review: on nurse-led out-patient coronary care; team-based collaborative coronary care  models; mHealth type telephony applications etc.) in the development of the questions and prompts. The paper needs a defense of the use of the author-generated, currently unreferenced interview topic guides as your ‘theoretical framework’ for deductive analysis.** **○ If this is not possible, could you include in a limitations section of the paper the potential for confirmation/researcher bias in the findings and their interpretation?** **○ Given the use of the interview topic guide as the theme for deductive analysis, without knowledge of the peer-reviewed evidence underlying the development of questions (and prompts) for the respective stakeholder categories, the issue of confirmation/researcher bias in the paper cannot be assessed. This link to the article “Issues of validity and reliability in qualitative research” may be of use in responding to these comments: https://ebn.bmj.com/content/18/2/34** **The assessment of the integrity of qualitative research requires evidence in the paper of the researchers’ awareness of potential (generally unconscious) bias in the research design and its analysis and reporting. I have included a link to the relevant JBI International Critical Appraisal Tool for Qualitative Research download page: https://jbi.global/critical-appraisal- tools - Could you review the overall checklist, with special attention to questions 6 and 7 (copied below) in the list and include relevant responses in your text?** **Is there a statement locating the researcher culturally or theoretically?**

**Is the influence of the researcher on the research, and vice- versa, addressed?**

 The interview questions and prompts were developed based on the proposed CCM as well as relevant literature on collaborative care models and its perceived barriers and facilitators. We have also added the positionality statement: Positionality of the Researcher The researcher who collected and analysed the data (SCT) is a trained nurse with a PhD in public health. She is also a trained qualitative researcher. Although her education and training could have an influence on the research, having not experienced the illness or the care seeking experience of the condition that was being researched, she is placed well to do the research. Being young female researcher and sharing the same cultural identity as that of the participants, helped her interact with the patients without them being fearful.  Nevertheless, being from the same institution and the statements about her association with the research centre may have had some influence on the research participants.

**6. The paper has reported that medication adherence was an issue from clinician perspectives but noted that this was not a theme in patient transcripts. Given the disproportionately small sample size of patients (8 of 22 participants) in the study, further evidence (e.g. from peer-reviewed studies on the subject) is required to support the conclusion. Further, as no patient reported non- adherence to medication, barriers to treatment compliance from the patient perspective could not be identified.**

 Thank you for pointing this out. In terms of general compliance, you were seeing patients who had already come to the hospital clinic, that means they are compliant in terms of visiting their doctors. Therefore, we might have got patients who might have been generally compliant. Additionally, patients can also perceive the researchers as part of the same hospital which could influence their response on treatment adherence.  We have added it in our limitations.

**7. It is recommended that a separate Limitations section be added to the paper and that this issue and others mentioned above be included under this heading. A recommendation for further research on patient perspectives of treatment non-adherence is also indicated.**

 We have added the limitations section to the paper.

# Reviewer report: Reviewer response for version 1

**DOI:** 10.21956/wellcomeopenres.23420.r87497

**Published:** 2024-07-16

**Authors:** GK Mini

**Affiliations:** 1 Global Institute of Public Health, Ananthapuri Hospitals and Research Institute (Ringgold ID: 75682), Trivandrum, Kerala, India

**Feasibility of a nurse-led, mHealth-assisted, and team-based collaborative care model for heart failure care in India: Findings from a multi-stakeholder qualitative study**

**Comments**

**General comments:**

- The study set out to investigate the practicality of a nurse-led and mHealth-supported approach to caring for individuals with heart failure (HF) in India (as reported at the end of the introduction section). However, the title refers to a "team-based collaborative care model". Additionally, the abstract aimed to obtain perceptions of potential challenges to care and suggestions on multiple components of the proposed CCM in managing HF and its feasibility. The conclusion mainly focuses on the obstacles in HF management and the collaborative, nurse-led care model. Please ensure that these concepts are presented consistently throughout the study.
- The manuscript lacks a clear establishment of the concept of a care model. Figure 1 summarizes the findings from the multi-stakeholder interviews, while Figure 2 illustrates the feasibility of a nurse-led mHealth-assisted CM for heart failure management. The model presented in Figure 2 emphasizes the implementation of mHealth-assisted nurse-led care for HF management. These findings indicate the promise of implementing a nurse-led strategy assisted by mHealth for HF management. The title's wording, such as "team-based collaborative care model," needs more conceptualization.

**Specific comments**

**In methods:**

- In the method section under the title "study setting" (second paragraph), the details presented about how the researchers identified the stakeholders from three private, one co-operative, and one public sector tertiary care hospital were not clearly discussed.
- Under the title "Study participants," it states, "We conducted in-depth interviews with all of them." Following that, it mentions, "We purposively selected cardiologists and nurses with experience in HF clinic." This requires clarification.
- In the same sub-title above, it was mentioned that "These centres were chosen based on whether they had dedicated HF clinics." However, in the result section under the title "Participant characteristics," it is indicated that out of these centres, the selected cooperative clinic did not have a dedicated HF clinic. This seems inconsistent.
- In the last paragraph under the title "Data collection, quality control, and management," it was mentioned that the interviews lasted for 30-45 minutes. However, it is important to note that the interviews with different targeted participants may have varied in duration. This variability needs to be described in detail.
- It was not clear how many participants were approached in the different groups.

**Results and Discussion:**

- Effective communication with doctors turned out to be a challenge due to language barriers. Based on the specific hospital and doctor surveyed, further details are required.
- The conclusion highlighted long-distance travel as a major obstacle. However, in the results and discussions, the main related issue was identified as financial constraints in traveling. While one doctor reported long-distance travel as an obstacle, it did not emerge as a concern from the patient's point of view. Therefore, the authors may need to revise the conclusion accordingly.

Is the work clearly and accurately presented and does it cite the current literature?

Yes

If applicable, is the statistical analysis and its interpretation appropriate?

Partly

Are all the source data underlying the results available to ensure full reproducibility?

Yes

Is the study design appropriate and is the work technically sound?

Yes

Are the conclusions drawn adequately supported by the results?

Yes

Are sufficient details of methods and analysis provided to allow replication by others?

Partly

Reviewer Expertise:

Public Health, Biostatistics

I confirm that I have read this submission and believe that I have an appropriate level of expertise to confirm that it is of an acceptable scientific standard, however I have significant reservations, as outlined above.

# Response

**Published:** 2024-10-10

**Authors:** Panniyammakal Jeemon

**Affiliations:** Sree Chitra Tirunal Institute for Medical Sciences and Technology, Trivandrum, India

Dear Madam, Thank you for reviewing our manuscript and for your thoughtful feedback.  We

 strongly believe that the comments and our subsequent revisions have greatly enhanced the quality of our manuscript. Please find below a point-by-

 point response to the reviewer's comments.

**1. The study set out to investigate the practicality of a nurse-led and mHealth-supported approach to caring for individuals with heart failure (HF) in India (as reported at the end of the introduction section). However, the title refers to a "team-based collaborative care model". Additionally, the abstract aimed to obtain perceptions of potential challenges to care and suggestions on multiple components of the proposed CCM in managing HF and its feasibility. The conclusion mainly focuses on the obstacles in HF management and the collaborative, nurse-led care model. Please ensure that these concepts are presented consistently throughout the study.**

 Thank you for pointing this out. This study is part of a larger intervention that aims to develop a team-based collaborative care model (CCM), facilitated by trained nurses, for managing HF with the support of mHealth and evaluate its acceptability and effectiveness in the Indian setting. This study aimed to obtain perceptions of potential challenges to heart failure care and suggestions on multiple components of the proposed CCM in managing HF and its feasibility. Since this model envisaged a nurse-facilitated CCM, we also were looking for the acceptability of nurses in leading this collaborative care.  We have reworded the objectives in the main text to reflect this. 

**2. The manuscript lacks a clear establishment of the concept of a care model. Figure 1 summarizes the findings from the multi-stakeholder interviews, while Figure 2 illustrates the feasibility of a nurse-led mHealth-assisted CM for heart failure management. The model presented in Figure 2 emphasizes the implementation of mHealth-assisted nurse-led care for HF management. These findings indicate the promise of implementing a nurse-led strategy assisted by mHealth for HF management. The title's wording, such as "team-based collaborative care model," needs more conceptualization.**

 Thank you for the comment. This study is part of a larger intervention that aims to develop a team-based collaborative care model (CCM), facilitated by trained nurses, for managing HF with the support of mHealth and evaluate its acceptability and effectiveness in the Indian setting. We conceptualized the team-based collaborative care model as a team-based approach to HF care with nurses as a key team player. We have added this to the manuscript.

**3. In the method section under the title "study setting" (second paragraph), the details presented about how the researchers identified the stakeholders from three private, one co-operative, and one public sector tertiary care hospital were not clearly discussed.**

 Thank you for pointing out this. This has been mentioned in the subsequent section on study participants, recruitment, and sampling: We purposively selected cardiologists and nurses with experience in HF care from participating centers of a large cluster randomized trial. We have purposefully selected the stakeholders from these centers based on their experience and expertise in heart failure management and their willingness to be interviewed.

**4. Under the title "Study participants," it states, "We conducted in-depth interviews with all of  them." Following that, it mentions, "We purposively selected cardiologists and nurses with experience in HF clinic." This requires clarification.**

 Thank you. “We conducted in-depth interviews with all of them."- Here we meant the method of data collection i.e., in-depth interview was conducted with all of the participants.

**5. In the same sub-title above, it was mentioned that "These centres were chosen based on whether they had dedicated HF clinics." However, in the result section under the title "Participant characteristics," it is indicated that out of these centres, the selected cooperative clinic did not have a dedicated HF clinic. This seems inconsistent.**

 Thank you for the comment. We have selected centers with dedicated HF clinics and those without HF clinics. We have re-phrased it as "These centres were chosen based on whether they had dedicated HF clinics or not”.

**6. In the last paragraph under the title "Data collection, quality control, and management," it was mentioned that the interviews lasted for 30-45 minutes. However, it is important to note that the interviews with different targeted participants may have varied in duration. This variability needs to be described in detail.**

 Thank you. Majority of the interviews lasted 30 minutes, with a few of them with some cardiologists and nurses, which was between 30-45 minutes. Therefore, we have mentioned 30-45 minutes. We have made the necessary changes in the manuscript. “Majority of the interviews lasted 30 minutes, with a few interviews with cardiologists and nurses, which lasted between 30-45 minutes.”

**7. It was not clear how many participants were approached in the different groups.**

 A total of 22 participants were interviewed for the study in different groups, and all of them consented to be interviewed. This has been mentioned in the manuscript in the section Participant Characteristics- All the participants approached for the interview consented to participate in the study.

**8. Effective communication with doctors turned out to be a challenge due to language barriers. Based on the specific hospital and doctor surveyed, further details are required.**

 These findings are based on the interviews with HF patients from a tertiary super-specialty hospital, where the treating doctors are from different parts of the country. Therefore, the patients experienced difficulties in communicating with doctors. Even though this experience is specific to one hospital, it is very important from the patient perspective in their continuity of care. We have added it in the manuscript.

**9.The conclusion highlighted long-distance travel as a major obstacle. However, in the results and discussions, the main related issue was identified as financial constraints in traveling. While one doctor reported long-distance travel as an obstacle, it did not emerge as a concern from the patient's point of view. Therefore, the authors may need to revise the conclusion accordingly.**

 Long-distance travel could also mean an additional financial burden for the patients with respect to travel and overnight stay. We have made corrections in the conclusion to include this.
